# COVID-19 vaccine acceptance and perceived risk among pregnant and non-pregnant adults in Cameroon, Africa

**DOI:** 10.1371/journal.pone.0274541

**Published:** 2022-09-13

**Authors:** Nuwan Gunawardhana, Kendall Baecher, Alexander Boutwell, Seraphine Pekwarake, Mirabelle Kifem, Mary Glory Ngong, Anthony Fondzeyuf, Gregory Halle-Ekane, Rahel Mbah, Pius Tih, Jodie Dionne-Odom, Denis M. Tebit

**Affiliations:** 1 Division of Infectious Diseases, Department of Medicine, University of Alabama at Birmingham, Birmingham, Alabama, United States of America; 2 School of Medicine, University of Alabama at Birmingham, Birmingham, Alabama, United States of America; 3 Cameroon Health Initiative at the University of Alabama at Birmingham (CHI UAB), Birmingham, Alabama, United States of America; 4 Department of Obstetrics and Gynecology, University of Buea, Buea, Cameroon; 5 Department of Microbiology, University of Venda, Thohoyandou, South Africa; 6 Global Biomed Laboratories Inc., Lynchburg, VA, United States of America; The University of Jordan, JORDAN

## Abstract

**Background:**

The public health response to the global COVID-19 pandemic has varied widely by region. In Africa, uptake of effective COVID-19 vaccines has been limited by accessibility and vaccine hesitancy. The aim of this study was to compare perceptions of COVID-19 infection and vaccination between pregnant women and non-pregnant adults in four regions of Cameroon, located in Central Africa.

**Methods:**

A cross-sectional survey study was conducted at urban and suburban hospital facilities in Cameroon. Participants were randomly selected from a convenience sample of adult pregnant and non-pregnant adults in outpatient clinical settings between June 1^st^ and July 14^th^, 2021. A confidential survey was administered in person by trained research nurses after obtaining written informed consent. Participants were asked about self-reported sociodemographics, medical comorbidities, perceptions of COVID-19 infection, and vaccination. Descriptive statistics were used for survey responses and univariate and multivariable logistic regression models were created to explore factors associated with COVID-19 vaccine acceptability.

**Results:**

Fewer than one-third of participants were interested in receiving the COVID-19 vaccine (31%, 257/835) and rates did not differ by pregnancy status. Overall, 43% of participants doubted vaccine efficacy, and 85% stated that the vaccine available in Africa was less effective than vaccine available in Europe. Factors independently associated with vaccine acceptability included having children (aOR = 1.5; p = 0.04) and higher education (aOR = 1.6 for secondary school vs primary/none; p = 0.03). Perceived risks of vaccination ranged from death (33%) to fetal harm (31%) to genetic changes (1%). Health care professionals were cited as the most trusted source for health information (82%, n = 681).

**Conclusion:**

COVID-19 vaccine hesitancy and misinformation in Cameroon was highly prevalent among pregnant and non-pregnant adults in 2021 while vaccine was available but not recommended for use in pregnancy. Based on study findings, consistent public health messaging from medical professionals about vaccine safety and efficacy and local production of vaccine are likely to improve acceptability.

## Introduction

COVID-19 vaccination is critical to curbing the global spread of the Severe Acute respiratory virus type 2 (SARS CoV-2) and reducing adverse outcomes of infection, including adverse birth outcomes. Vaccine uptake differs greatly by region. In Africa, uptake has been challenged by limited access during the early phases of the pandemic response, supply chain issues, and low levels of vaccine acceptance [1]. As of February 2022, COVID-19 vaccine uptake in Africa was 29% (12% fully vaccinated and 17% partially vaccinated) with the highest rates in Seychelles (77%), Mauritius (72%), Morocco (63%), Rwanda (56%), and Tunisia (39%). Current vaccination rates in many countries are <10%, including Cameroon in Central Africa with one of the lowest global vaccination rates at 3.2% [2]. Cameroon is also one of four African countries (and 16 globally) where COVID-19 vaccination in pregnancy is not recommended.

Perception of the COVID-19 vaccine is an important determinant of acceptability [3, 4]. A recent survey study conducted in 15 African countries found high willingness (93–94%) in Ethiopia and Niger but moderate willingness (59% and 65%) in Senegal and the Democratic Republic of Congo [5]. In Cameroon, a survey study conducted prior to the availability of vaccine showed acceptability of 15% [6]. Reasons for vaccine hesitancy in Africa are complex and concerns about vaccine safety (including risk during pregnancy and on fertility), effectiveness, and lack of trust in the government have been noted [7]. COVID-19 vaccine acceptability also differs according to pregnancy status. In a global survey conducted in 2020 that asked participants to assume the availability of COVID-19 vaccine with 90% efficacy, 52% of pregnant women and 73% of non-pregnant women were willing to be vaccinated [8].

According to the World Health Organization (WHO) COVID-19 dashboard, there were 106,000 confirmed cases of COVID-19 in Cameroon and 1,770 deaths as of November 2021 [9]. Despite the availability of COVID-19 vaccine in Cameroon, only 607, 000 doses had been administered at that time (1% of a population of 28 million). We designed a comparative study aimed at understanding COVID-19 vaccine acceptance and hesitancy among pregnant women and non-pregnant adults in the setting of vaccine availability. We hypothesized that vaccine acceptability would be lower in pregnant women and sought to identify trusted sources of health information to support future public health efforts.

## Methods

### Study design and participants

A cross-sectional survey study was conducted at hospital facilities in four of the ten regions of Cameroon between July 1^st^ and August 14^th^, 2021. During this period, COVID-19 vaccine was recommended and encouraged by the ministry of health and regional public authorities for adults, but vaccination was not recommended for pregnant women. Two different vaccine types manufactured by AstraZeneca and Sinopharm were available at a number of sites across the country. By design, the study sample selection aimed for equal representation of pregnant women and a comparison group of nonpregnant adults who were 18 years and older. Potential subjects who were cognitively impaired, residing in institutions such as prisons and schools, those in emergency or life-threatening situations, and those with any language barrier unable to communicate (illiterate, dysphasic) with the researcher were excluded. Recruitment took place among randomly selected patients and pregnant subjects seeking outpatient care at four urban and suburban facilities: Nkwen Baptist Hospital in Bamenda (NBHB); Baptist Hospital Mutengene (BHM), Mboppi Baptist Hospital, Douala (MBHD), and Etoug-Ebe Baptist Hospital, Yaoundé (EBHY). These four facilities are all run by the Cameroon Baptist Convention Health Board.

The anonymous survey was administered verbally in a private room to participants in English, Pidgin English, or French by trained research nurses at the Cameroon Health Initiative at the University of Alabama, Birmingham (CHI UAB) after informed consent was provided and documented. The consent form and the questionnaire were written such that a primary school reader could understand. Survey information was collected on paper and information was entered into an electronic database.

### Survey measures

The survey was adapted from a previously developed questionnaire designed to assess COVID-19 vaccine acceptability in Cameroon and published by Dinga et al [6]. Participants responded to a series of yes or no and Likert scale questions, multiple-choice, and few free text responses to collect quantitative and qualitative data on vaccine perceptions. The survey had 21 questions and took 10–15 minutes to administer. Survey sections included sociodemographic information, medical comorbidities, perception of COVID-19 infection and risk of infection, likelihood of COVID-19 vaccination for self and for children, barriers to vaccination, and preferred source of medical information. Perceptions of COVID-19 vaccine were assessed with the question: “Have you heard of any risks associated with COVID-19 vaccination? If yes, please cite them.” A copy of the study questionnaire is provided in the supplementary data section.

### Study outcomes

The primary study outcome was COVID-19 vaccine acceptability defined as a positive response to the question “If you were offered a COVID-19 vaccine today, would you take it?” This is a standard survey question that has been used to assess vaccine acceptability in other published studies [10]. There were four possible responses in a Likert scale to assess the strength of acceptability: definitely yes; maybe; not sure; definitely no. We also determined perceptions of COVID-19 vaccination safety and efficacy stratified by pregnancy status and elicited detailed description of the perceived risks of vaccination.

### Statistical analysis

Survey data was categorized by response and stratified by pregnancy status to compare the two groups. The Chi-Square test was used to compare the categorical variables. Median values and interquartile ranges were calculated for continuous variables. In order to assess potential factors associated with vaccine acceptability, we created univariate and multivariable models using logistic regression for the dichotomous outcome and included demographic factors associated with vaccine uptake in previously published studies (age, gender, pregnancy status, educational status, presence of comorbid conditions associated with severe COVID-19 outcomes), and three non-demographic variables: awareness of COVID-19, personal knowledge of a person with COVID-19 infection, and history of vaccination against other diseases. We created a dichotomous primary outcome for vaccine acceptability by combining “definitely yes” with “maybe” as acceptable and “not sure” with “definitely no” as not acceptable. The same factors were included in unadjusted and adjusted models to calculate odds ratios and 95% confidence intervals and statistical significance was set at p<0.05. We calculated that a sample size of 752 persons would allow us to detect a 10%-point difference in vaccine acceptability between pregnant and non-pregnant adults, assuming 85% power and alpha level of 0.05. Statistical analysis was performed using R version 4.1.2.

### Ethical considerations

This study was reviewed and approved by the Institutional Review Board of the Cameroon Baptist Convention Health Board with study approval number IRB2021-33. Participants provided written informed consent prior to completion of the survey.

## Results

### Participant characteristics

A total of 835 participants were surveyed, with ages ranging from 17 to 78 years (median age 29). Consistent with population characteristics in Cameroon, younger adults (age 17–29) comprised 52% of the study population and 6% were above age 50 (Table 1). Less than half of survey respondents were pregnant (n = 385; 46%), most were female (n = 705; 84%) and 67% had children. Pregnant women were younger (median age 27 vs 31 years) and had higher educational levels compared to non-pregnant adults (44% vs 34% with a university degree; p<0.001). Most participants (61%) were surveyed in urban Yaoundé or Douala and the remainder had visited facilities in suburban regions of northwest and southwest Cameroon. Medical comorbidities were reported by a minority of participants (4% of pregnant women and 14% of non-pregnant participants; p = 0.02) and 70% reported having received other (non-COVID-19) vaccinations in the past (Table 1).

**Table 1:** Participant characteristics (n = 835).

	Pregnant	Non-pregnant	Total
	n (%)	n (%)	n (%)
	n = 387	n = 448	n = 835
**Median Age in years [IQR]**	27 [24, 31]	31 [26, 40]	29 [25, 35]
**Age Categories**			
17–29	255 (65.9)	181 (40.4)	436 (52.2)
30–39	122 (31.5)	152 (33.9)	274 (32.8)
40–49	10 (2.6)	68 (15.2)	78 (9.3)
50+	0 (0.0)	47 (10.5)	47 (5.6)
**Gender**			
Female	387 (100)	318 (71.0)	705 (84.4)
Male	0	130 (29.0)	130 (15.6)
**Highest Level of Education (n = 832)**			
None/Primary	50 (13.0)	101 (22.5)	151 (18.1)
Secondary	168 (43.4)	194 (43.3)	362 (43.4)
University	168 (43.4)	151 (33.7)	319 (38.2)
**Facility Location**			
Bamenda (suburban Northwest)	74 (19.1)	76 (17.0)	150 (18.0)
Douala (urban Littoral)	125 (32.3)	127 (28.3)	252 (30.2)
Mutengene (suburban Southwest)	64 (16.5)	111 (24.8)	175 (21.0)
Yaoundé (urban Central)	124 (32.0)	134 (29.9)	258 (30.9)
**Prevalent Medical Comorbidities**			
Any Condition	16 (4.1)	61 (13.6)	77 (9.2)
Cardiovascular Disease	0 (0.0)	18 (4.02)	18 (2.2)
Pulmonary Disease	5 (1.3)	9 (2.0)	14 (1.7)
Infectious Diseases	6 (1.6)	8 (1.8)	14 (1.7)
Diabetes or Cancer	0 (0.0)	6 (1.3)	6 (0.7)
Other*	5 (1.3)	20 (4.5)	25 (2.9)
**Has received non-COVID vaccines**	269 (69.5)	314 (70.1)	583 (69.8)
**Has living children**	258 (66.7)	331 (73.9)	589 (70.5)
**Children have received other vaccines**	199/258 (77.1)	289/331 (87.3)	488/589 (82.9)

*Other: anemia, arthritis, epilepsy, vision changes

### Covid-19 awareness, vaccine perceptions and trusted sources of health information

Nearly all respondents (95%) had heard of COVID-19, and 80% believed that the virus was circulating in Cameroon at the time of survey in mid-2021 (Table 2). Most (88%) were aware of the COVID-19 vaccine and 54% reported that COVID-19 vaccine was available in their region of residence. Participants were more worried about friends and family getting COVID-19 than themselves (73% vs 63%). Pregnant women were less likely to know someone with COVID-19 compared to non-pregnant adults (21% vs 31%; p<0.01). Only 27% of respondents reported COVID-19 testing in the past (self-reported positivity rate 4% in pregnant women vs 15% of non-pregnant adults; p = 0.03).

**Table 2:** COVID-19 awareness, perceptions and vaccine acceptability by pregnancy status.

	Pregnant	Non-pregnant	Total	p-value
	n (%)	n (%)	n (%)	
	n = 387	n = 448	n = 835	
**Has heard of COVID-19**	360 (93.0)	432 (96.4)	792 (94.9)	**0.04**
**Has heard of COVID-19 vaccine**	336 (86.8)	397 (88.6)	733 (87.9)	0.44
**Agrees that COVID-19 is in Cameroon**	309 (79.8)	352 (78.6)	661 (79.2)	0.64
**Worried about COVID-19 in self**	239 (61.8)	289 (64.5)	528 (63.2)	0.38
**Worried about COVID-19 in friends/family**	275 (71.1)	334 (74.6)	609 (72.9)	0.42
**Knows someone who had COVID-19**	80 (20.7)	139 (31.0)	219 (26.2)	**<0.01**
**Thinks the COVID-19 Vaccine Works**				**0.04**
Yes	132 (38.3)	135 (33.1)	267 (35.5)	
Not sure	62 (18.0)	104 (25.5)	166 (22.0)	
No	151 (43.8)	169 (41.4)	320 (42.5)	
**COVID-19 Vaccine Acceptability for Self**				0.11
Definitely Yes	36 (9.3)	45 (10.0)	81 (9.7)	
Maybe	72 (18.6)	104 (23.2)	176 (21.1)	
Not Sure	99 (25.6)	95 (21.2)	194 (23.2)	
Definitely No	180 (46.5)	204 (45.5)	384 (46.0)	
COVID-19 Vaccine Acceptability for Children**				0.42
Definitely Yes	37 (9.6)	44 (9.8)	81 (9.7)	
Maybe	63 (16.3)	86 (19.2)	149 (17.8)	
Not Sure	93 (24.0)	115 (25.7)	208 (24.9)	
Definitely No	189 (48.8)	202 (45.1)	391 (46.8)	
**Vaccine Acceptability if Produced in Africa**				0.98
Definitely Yes	102 (26.4)	109 (24.3)	211 (25.3)	
Maybe	111 (28.7)	119 (26.6)	230 (27.5)	
Not Sure	84 (21.7)	112 (25.0)	196 (23.5)	
Definitely Not	90 (23.3)	108 (24.1)	198 (23.7)	
**Top Source for Health Information**				0.11
Hospital or Medical Clinic	274 (70.8)	310 (69.2)	584 (69.9)	
Radio	42 (10.9)	50 (11.2)	92 (11.0)	
Social Media	18 (4.7)	27 (6.0)	45 (5.4)	
Ministry of Health	16 (4.1)	17 (3.8)	33 (4.0)	
School	16 (4.1)	11 (2.5)	27 (3.2)	
Newspapers or Books	13 (3.4)	10 (2.2)	23 (2.8)	
Friends or Family	6 (1.6)	8 (1.8)	14 (1.7)	
Church or Ministry	0 (0.0)	9 (2.0)	9 (1.1)	
Unspecified	2 (0.5)	6 (1.3)	8 (1.0)	

*Among n = 589 with children

Pregnant women were more likely than non-pregnant adults to agree that the COVID-19 vaccine works (38% vs 33%; p = 0.04). When asked whether they would accept a COVID-19 vaccine if it was available and offered today, 46% of participants responded “definitely no”, 10% responded “definitely yes” and 44% responded “not sure” or “maybe”. Interest in vaccination was similar between pregnant and non-pregnant respondents (p = 0.11) and rates of acceptability for COVID-19 vaccination in children were similar. A majority of participants (53%, n = 441) said that they would be more likely to accept a COVID-19 vaccine if it had been produced and distributed within Africa.

**Fig 1:**
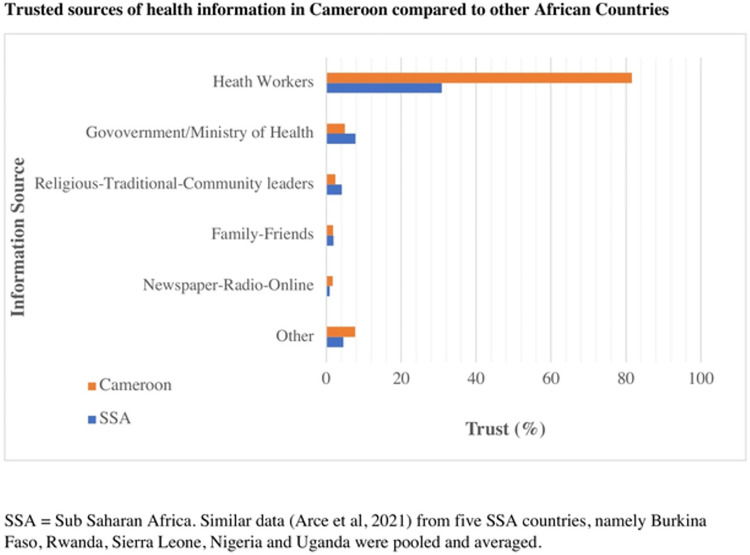
Trusted sources of health information in Cameroon compared to other African countries.

Two in three respondents (66%) thought that more information would be useful to those with concerns about COVID-19 vaccination. The top three sources of health information cited by participants were: healthcare workers in facilities (70%), radio (11%) and social media (5%). The most trusted sources of health information were healthcare professionals, cited by 82% (n = 681). Other top sources included scientists (7%), and ministry of health officials (5%). Fig 1 compares these trusted sources between Cameroonians surveyed and other sub-Saharan countries in similar published studies.

### Perceptions of COVID-19 vaccination

**Fig 2:**
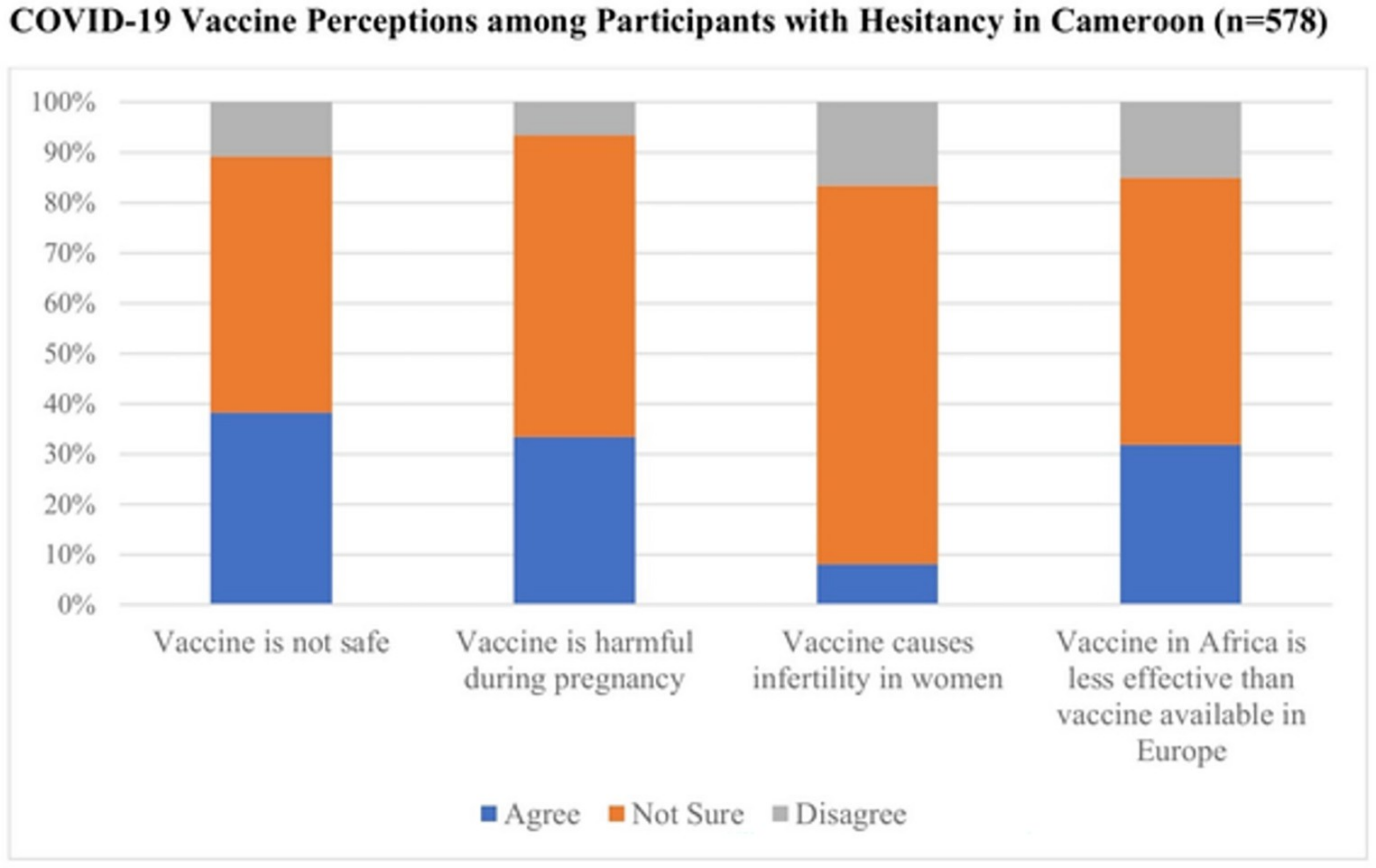
COVID-19 vaccine perceptions among participants with hesitancy in Cameroon (n = 578).

Both pregnant and non-pregnant participants in Cameroon reported great uncertainty about COVID-19 vaccine safety. Most expressed uncertainty about vaccine efficacy (55%), vaccine safety during pregnancy (61%), and the impact of vaccination on fertility (73%). When specifically asked if the COVID-19 vaccine could cause infertility, fewer pregnant women (5%; n = 19) agreed than non-pregnant adults (31%; n = 139; p = 0.03). Many respondents stated concern that the vaccine could cause fetal harm during pregnancy (31%, n = 256). When asked if the COVID-19 vaccine in Africa was less effective than the COVID-19 vaccine in Europe, 30% agreed, 15% disagreed, and 55% were not sure. Vaccine perceptions among respondents with COVID-19 vaccine hesitancy (n = 578) is presented in Fig 2.

**Fig 3:**
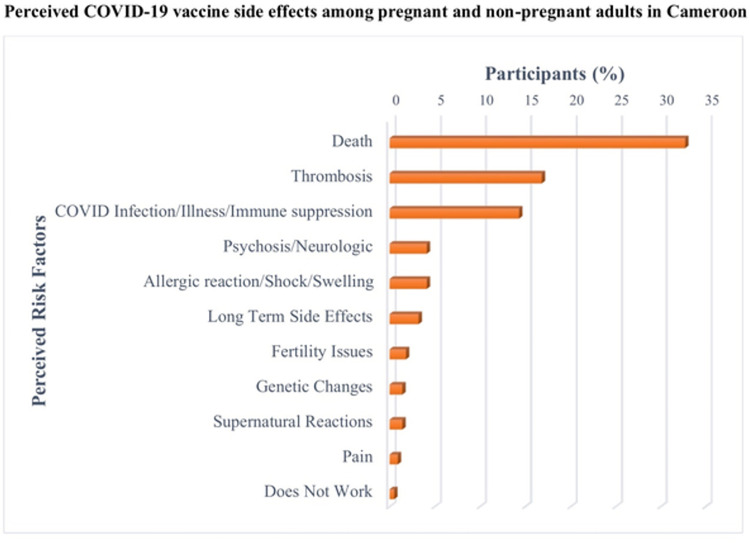
Perceived COVID-19 vaccine side effects among pregnant and non-pregnant adults in Cameroon.

Fig 3 demonstrates specific adverse outcomes attributed to COVID-19 vaccination that survey respondents were aware of. The five most common outcomes cited were death (33%; n = 72), thrombosis, (17%; n = 37), illness (6%; n = 14), COVID-19 infection (6%; n = 12) and long-term side effects (3%; n = 7). Twice as many subjects in the non-pregnant group considered death as a risk factor for COVID-19 vaccination than in the pregnant group (42% vs 22%). Only 2% (n = 4) cited concerns about infertility. Other perceived risks cited by fewer than 2% included immunosuppression, swelling, transformation into a robot and microchip implantation.

### Factors associated with COVID-19 vaccine acceptability

In the unadjusted model, factors associated with vaccine acceptability included having children (OR 1.5, 95% CI 1.1–2.1; p = 0.02), secondary education levels compared to primary education or less (OR 1.4, 95% CI 1.0–2.2; p = 0.03) and awareness of COVID-19 (OR 2.4, p = 0.04) (Table 3). The association between pregnancy status and lower vaccine acceptability was not statistically significant (OR = 0.8, 95% CI 0.6–1.0; p = 0.1). In the adjusted model, having children (aOR = 1.5, 95% CI 1.03–2.2; p = 0.04) and secondary education (aOR = 1.6, 95% CI 1.04–2.5; p = 0.03) were independently associated with vaccine acceptability.

**Table 3:** Factors associated with COVID-19 vaccine acceptability in Cameroon.

Factor	Unadjusted OR (95% CI)	p-value	Adjusted OR (95% CI)	p-value
Age >30 years	1.12 (0.84–1.51)	0.436	0.91 (0.65–1.28)	0.591
Male gender	1.39 (0.93–2.05)	0.100	1.29 (0.83–2.0)	0.260
Has children	1.48 (1.06–2.09)	0.022	1.49 (1.03–2.17)	0.036
Currently pregnant	0.78 (0.58–1.04)	0.095	0.86 (0.61–1.21)	0.394
Education		0.034		0.027
None/Primary	REF		REF	
Secondary	1.44 (0.95–2.19)		1.59 (1.04–2.46)	
University	0.96 (0.62–1.49)		1.08 (0.68–1.71)	
Has medical comorbidities	1.24 (0.75–2.01)	0.393	1.15 (0.68–1.92)	0.596
Knows what COVID-19 is	2.37 (1.11–5.88)	0.040	2.26 (1.03–5.68)	0.058
Knows someone with COVID-19	0.93 (0.66–1.30)	0.627	0.93 (0.65–1.33)	0.703
Received other vaccines in the past	0.91 (0.66–1.25)	0.551	0.90 (0.65–1.25)	0.520

## Discussion

Our survey showed a persistent lack of acceptability of the COVID-19 vaccine in Cameroon among pregnant women and non-pregnant adults surveyed in mid-2021. Many participants cited doubts about vaccine efficacy, concerns about vaccine safety (including death) and harms when administered during pregnancy. Vaccines remain the most effective intervention to prevent COVID-19 infection and WHO classifies vaccine hesitancy as one of the main threats affecting global health [6]. Understanding vaccine hesitancy and addressing specific and common concerns is necessary to increase global vaccine uptake.

In Cameroon, COVID-19 vaccine acceptability in 2021 was only 31%. This was higher than the baseline rate of 15% acceptability documented in a similar survey conducted in country before vaccine availability [6]. This suggests that COVID-19 vaccine sensitization and information in Cameroon had some benefit. However, vaccine acceptance in Cameroon is an outlier compared to other LMICs where acceptance averages 80% [10, 11]. Consistent with these findings, recent data indicate that only 3% of the Cameroonian population had received the COVID-19 vaccine as of February 2022 [2]. Although this number has increased to 5%, it remains one of the lowest vaccination rates in the region and it has been more challenging to manage and control the national COVID-19 pandemic in a country with limited health resources and medical personnel [12].

In order to understand what is unique about the factors driving COVID-19 vaccine hesitancy in Cameroon, we compared the results of our multivariable model with other studies. Higher education and having children were independently associated with vaccine acceptability in our study and others. Respondents with a secondary education were significantly more likely to take the vaccine compared to those with a primary and university education. The lower level of acceptability among those with university level education may be due to increased skepticism about circulating COVID-19 information [3, 13]. This has been documented in other African countries but in other countries, higher education is more consistently associated with vaccine acceptance [10, 13–17]. Unlike other studies, and in contrast to our hypothesis, pregnant women had similar acceptability rates compared to non-pregnant adults. This was particularly surprising since the government policy at the time of the survey excluded pregnant women from those eligible to receive COVID-19 vaccine. Although the US CDC recommended universal COVID-19 vaccination of pregnant women in 2021, national guidelines in Cameroon recommending vaccination in pregnancy were not adopted until February 2022. Unfortunately, the new policy is only inclusive of Pfizer/BioNTech mRNA vaccines that are not widely available in the country. In terms of vaccine acceptability by pregnancy status, we suspect that all respondents were receiving the same information about vaccination safety and efficacy which contributed to similar rates of hesitancy. On the contrary, recent COVID-19 acceptance studies among pregnant women in the United Kingdom, United States and Romania showed relative higher rates of 50–80%, about 2–3 times higher than observed in our study [18–20]. The reasons for hesitancy in these studies included lack of access to health care facilities, trusting social media rumors or not believing in COVID-19 vaccines. Aligned with studies in LMIC settings showing variable results, we did not find a significant difference in vaccine acceptance according to age, gender, or personal knowledge of someone with COVID-19 [10].

Misinformation about COVID-19 and vaccination was pervasive. Nearly one in five respondents did not believe that COVID-19 was circulating in Cameroon despite weekly reports of confirmed cases and hospitalizations reported publicly by the ministry of health. Testing was limited in Cameroon at the time of the survey and the vast majority had never been tested for COVID-19 which likely contributed to lack of awareness of circulating infection in local households and communities.

The types of misinformation cited about vaccine risks were myriad and a few consistent patterns emerged–concerns about risk of death, thrombosis, infection/immune suppression, and long-term side effects. Each of these can be addressed with messaging about vaccine safety and the track record of millions of doses administered to date. Although infertility concerns have been cited in other studies of COVID-19 vaccine hesitancy this was not a major concern in our study [21]. This highlights that vaccine hesitancy is multifaceted and underlying concerns vary from one country to another [3, 22].

Most people surveyed had doubt about the reliability and effectiveness of the vaccine, similar to the earlier study by Dinga et al [6]. Since one third of respondents believed that the vaccine used in Europe was more effective that what is being sent to Africa, trust may be higher for COVID-19 vaccines that are manufactured in Africa. The slow introduction of COVID-19 vaccines in African countries gave time for hesitancy to gain a strong foothold and for conspiracies to mature [23, 24]. Public concerns were strengthened when some countries were forced to destroy hundreds of thousands of vaccine doses which expired shortly after arrival [25]. About 30% of donated vaccines were wasted in some low- and middle-income countries very close to the 35% of vaccines which had still not been utilized by February 2022 in some LMICs [26, 27]. Our study’s findings strengthen the call for pharmaceutical companies to establish manufacturing centers in Africa and share intellectual property rights to expand access to vaccination. This exciting initiative is now underway with mRNA vaccine plants being built in six African countries [28]. This will improve infrastructure and the capacity for young African scientists to produce vaccines that target new SARS-CoV-2 variants and other infections that lead to a disproportionate burden of disease in Africa.

A multi-pronged approach will be needed to tackle vaccine hesitancy in Cameroon and the national policy against vaccination in pregnancy should be reconsidered. This should include policy makers, healthcare workers, socio-cultural groups, researchers, advocates, and community/religious leaders coming together to discuss good policy proposals which should be transparent and provide regular updates so that the public is on the same page with these policymakers [29]. Health professionals were the most trusted and could play a vital role in encouraging vaccination if they themselves accept to take the vaccine. In a WHO survey done in November of 2021, only 25% of health care workers in 25 African countries were willing to take the vaccine compared to 80% in 22 high income countries [30, 31]. Incentives have also been effective in promoting vaccine acceptance in countries like Nigeria and India [10, 32]. Most participants stated that additional information about COVID-19 would increase vaccine uptake. Improved communication and messaging from informed healthcare professionals is likely to be highly impactful since they are the most trusted source of health information in Cameroon and other countries [33, 34]. Messaging through music, short drama sketches in public spaces, on the radio, and on social media would have broad reach. Some of these approaches discussed here are culturally relevant and are also mentioned among the six WHO and Lancet Commission recommendations to remediate COVID-19 vaccine resistance in sub-Saharan Africa [31].

The limitations of this study include the fact that it covered only four of ten regions in Cameroon. Cultural difference in a diverse society like Cameroon especially between the north and south of the country could lead to differences in vaccine hesitancy. However, this can only be proven in a more extensive study that covers the entire country. Timing was also a limiting factor as this study was done when only two vaccine types were available in the country. It is possible that broader vaccine choice could increase acceptance in the population although one could argue that it might not be the case given that these vaccines are all made “out of Africa”. The lack of safety data from vaccinated individuals in the Cameroon setting could also be a reason for this low acceptance.

## Conclusions

Vaccine acceptability rates in Cameroon among pregnant women and non-pregnant adults are among the lowest in the world. Many people cited misinformation about COVID-19 and expressed concerns about vaccine safety and efficacy. Low trust in vaccine campaigns led by the government in Cameroon and widely circulating inaccurate information about COVID-19 and vaccination may be critical reasons underlying vaccine hesitancy. High trust in medical professionals, uncertainty about potential outcomes, and significant interest in COVID-19 vaccine produced in Africa suggests a path forward. In order to reduce the impact of the COVID-19 pandemic, vaccine hesitancy must be addressed head-on.

## Supporting information

S1 File (PDF)

S2 File (XLSX)
