# Supplementary material for: COVID-19 vaccine acceptance and perceived risk among pregnant and non-pregnant adults in Cameroon, Africa
Source: PLoS One. 2022 Sep 13;17(9):e0274541. doi: 10.1371/journal.pone.0274541 (PMC9469991; doi:10.1371/journal.pone.0274541)
Supplement: S1 File — (PDF) [file pone.0274541.s001.pdf]

Survey about COVID-19 Vaccination in Cameroon

Date of Survey (DD/MM/YY) ________________ Initials of Person Administering Survey________________

+++

1) Personal information:
   a. Age (in years) ______________
   b. Gender (Male/Female) (circle one)
   c. Town of residence____________ (write in)
   d. Highest level of Educational completed (none, Primary, Secondary, University) (circle one)
   e. Are you currently pregnant (YES/NO)
2) Do you have any long-term medical problems? (YES/NO) (such as cancer, diabetes, heart disease, high blood pressure, asthma, anemia, other problem) (if yes, circle all that apply)
3) Have you ever heard of Coronavirus-19 or COVID-19? (YES/NO)
4) Do you think COVID-19 is in Cameroon? (YES/NO)
5) Have you had a COVID-19 test? (YES/NO)
   a. If yes, what was the result? (POSITIVE/NEGATIVE/AWAITING RESULTS)
6) Do you know anyone who has or who had COVID-19 infection? (YES/NO)
7) Do you know anyone who died of COVID-19? (YES/NO)
8) Are you worried about getting COVID-19? (YES/NO)
9) Are you worried about your family or friends getting COVID-19? (YES/NO)
10) Have you ever been vaccinated for any type of illness in the past? (YES/NO)
11) Do you have children? (YES/NO)
    a. If you have children, have they ever received any vaccines for any type of illness? (YES/NO)
12) Have you heard of a vaccine for COVID-19? (YES/NO)
    a. If yes, do you think the COVID-19 vaccine works? (YES/NO)
    b. If yes, have you heard of any risks of the COVID-19 vaccine? (YES/NO)
       i. (if YES, write in) ______________________
13) Is COVID-19 vaccine currently available anywhere in your region? (YES/NO)
14) If you were offered a COVID-19 vaccine today, would you take it? (circle one on the scale below)

    a) Definitely Yes b) Maybe c) Not sure d) Definitely No
15) If you have children, would you accept a COVID-19 vaccine for them if it was available today? (circle one)

    a) Definitely Yes b) Maybe c) Not sure d) Definitely No

16) If the COVID-19 vaccine was produced in Africa, would you be more likely to take it? (circle one)

a) Definitely Yes b) Maybe c) Not sure d) Definitely No

17) Do you think that more information would encourage people in Cameroon to take the vaccine? (circle)

a) Definitely Yes b) Maybe c) Not sure d) Definitely No

18) If you think more information may help, what type of information would be most helpful? (choose any)

a. what is in the vaccine
b. how the vaccine works
c. vaccine safety and vaccine side effects
d. the company and country where the vaccine was made
e. other information (please list) ____________________

19) Where do you get your information about health? (list top 3 in order as 1, 2, 3)

a. At the hospital or medical clinic ______
b. At school ________
c. Ministry of Health Resources ________
d. Newspapers and books ________
e. Other (please list) ________
f. Radio __________
g. Talking to Friends and Family ________
h. Social Media ___________
i. Pastor/Priest/Clergy ______
j. Herbalist/Traditional Healer______

20) Who do you trust the most for providing truthful information about health for you and your family? (list top 3 in order as 1, 2, 3)

a. Doctors and/or nurses ____________
b. Scientists ____________
c. Ministry of Health Officials ________
d. Community Leaders _____________
e. Journalists ____________
f. Herbalist/Traditional Healer______
g. Friends and Family ____________
h. Pastor/Priest/Clergy ___________
i. Other_____________

21) Do you agree or disagree with these four statements (circle one)

a. The COVID-19 vaccine in Africa is not as effective as the COVID-19 vaccine in Europe (AGREE/DISAGREE/NOT SURE)
b. The COVID-19 vaccine causes infertility in women (AGREE/DISAGREE/NOT SURE)
c. The COVID-19 vaccine available in Cameroon is not safe to take (AGREE/DISAGREE/NOT SURE)
d. Receiving the COVID-19 vaccine during pregnancy can cause harm to the baby (AGREE/DISAGREE/NOT SURE)

Thank you for taking the time to answer these questions today. We are grateful for your time and your responses to help us improve health in Cameroon!
